# Experimental Study on the Effect of Carbonation Reaction on the Properties of Imitation Site Soil

**DOI:** 10.3390/ma15082958

**Published:** 2022-04-18

**Authors:** Jianwei Yue, Xue Yang, Limin Zhao, Qingmei Kong, Ying Chen, Xuanjia Huang, Can Ma, Huicong Su, Wenhao Li, Huijie Gao

**Affiliations:** 1 School of Civil Engineering and Architecture, Henan University, Kaifeng 475004, China; yjw@vip.henu.edu.cn (J.Y.); yx1391989351@126.com (X.Y.); 104754190856@henu.edu.cn (Y.C.); hxj_henu@126.com (X.H.); 104753200696@henu.edu.cn (C.M.); a2104939923@163.com (H.S.); l308727900@126.com (W.L.); g18533929521@163.com (H.G.); 2 Key Laboratory for Restoration and Safety Evaluation of Immovable Cultural Relics in Kaifeng City, Kaifeng 475004, China

**Keywords:** imitation ruins, carbonation reaction, disintegration, shear strength, particle-size distribution

## Abstract

In this study, sodium methylsilicate and lime were selected to prepare the same proportion of Imitation Site Soil, and according to the principle of carbonation reaction of restoration materials, the effect of carbonation reaction on the performance of restoration soil of earthen sites was studied. The study has good significance for the conservation and restoration of earthen sites. The samples were cured with CO_2_ concentration and curing age as variables. After curing, the samples were tested to determine their water-resistant properties, uniaxial compressive strength, and pH value and a micro scanning electron microscope was used. The results indicated that the carbonation reaction can quickly improve the water resistance and compressive strength of imitation site soil, and reduced the water absorption by 16.67% compared to the specimens conditioned at 0.03% CO_2_ concentration. The UCS of specimens at 5%, 10%, and 15% CO_2_ concentrations increased by 72.22%, 131.19%, and 219.27%, respectively, compared with those at 0.03% CO_2_ concentration after the specimens were environmentally maintained in the carbonation chamber at 0.03%, 5%, 10%, and 15% CO_2_ concentrations for 120 h, respectively. The internal particle gradation of the imitation site soil improved after carbonation. These results provide a basis for improving the restoration technology of earthen sites.

## 1. Introduction

Earthen sites are an important carrier of civilization transmission, and they provide rich historical and cultural connotations and contain non-renewable information. Some earthen sites collapse and disappear due to weathering by wind, sun, rain, and other environmental effects. In such situations, many scholars have performed reinforcement research on earthen archaeological sites [1,2,3,4]. The research results have promoted studies on improving the earthen archaeological sites to a certain extent. Owing to the differences in the materials and environment considered in strengthening earthen archaeological sites, some earth sites offer problems such as rainwater scouring, fall off, and color difference after reinforcement. The subjective reason is that some researchers have insufficient understanding of the composition, formation mechanism, and mechanical properties of the earthen archaeological site. The fundamental reason is the basic characteristics, preparation technology, adaptive conditions, and reinforcement technology of the reinforcement materials [5,6].

**Figure 1:**
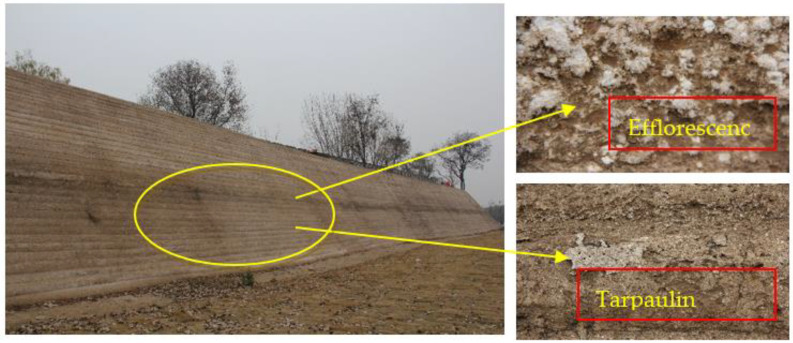
Deterioration of the Kaifeng City Wall.

In ancient times, lime, glutinous rice slurry, river sand, pottery fragments, or other materials were widely used and mixed with soil to form a triad to improve the strength and durability of the earthen archaeological site. Lime can be employed as a remediation material [7,8] because it can react with water and CO_2_ to produce Ca(OH)_2_ and CaCO_3_ to enhance soil cohesion between particles [9,10,11]. However, the carbonation reaction of lime in the natural environment is slow. Relevant studies have reported that it takes 240 days for lime to be completely carbonized into calcium carbonate [12]. Faster carbonization of lime and other materials under rich CO_2_ conditions had a better performance than naturally cured materials [13,14]. After repairing and reinforcing the existing earthen archaeological site, lime cannot completely carbonize into CaCO_3_, thereby resulting in surface cracking, peeling off, and alkali flashes in varying degrees due to the influence of the surrounding environment and efflorescence. As shown in Figure 1, the surface of the Kaifeng City Wall, which was newly repaired in 2020, has been damaged through efflorescence and surface upwarping, indicating defects in the technology used to repair the Kaifeng City Wall. In addition, domestic and foreign studies have shown that capillary water can affect soil strength and reduce resistance to wind erosion [15,16,17]. Accelerating the carbonation reaction of restored earthen ruins and improving their water-resistant properties are essential for the consolidation of earthen ruins. Therefore, when reinforcing the earthen sites, it is not only necessary to improve their mechanical properties, but also to consider the impermeability and water resistance [2,18,19,20]. Sodium methylsilicate (CH_3_Si(OH)_2_ONa) is an inorganic chemical waterproof material with excellent waterproof performance. It is widely used in waterproof, hydrophobic, material protection, and other fields [21,22].

This paper considers the Kaifeng City Wall soil as the object of study and configures the imitation earthen archaeological site accordingly. The imitation earthen archaeological site is reinforced with sodium methylsilicate and lime, and the sample is cured with CO_2_ concentration and curing time as variables. A disintegration test, uniaxial compression test, pH value, and micro scanning electron microscope test were performed on the cured samples to explore the effects of different curing conditions on the restoration and protection of the imitation earthen archaeological site in order to provide technical support for the restoration of the earthen archaeological site.

## 2. Materials and Methods

### 2.1. Preparation of Soil Samples and Stabilizers

**Figure 2:**
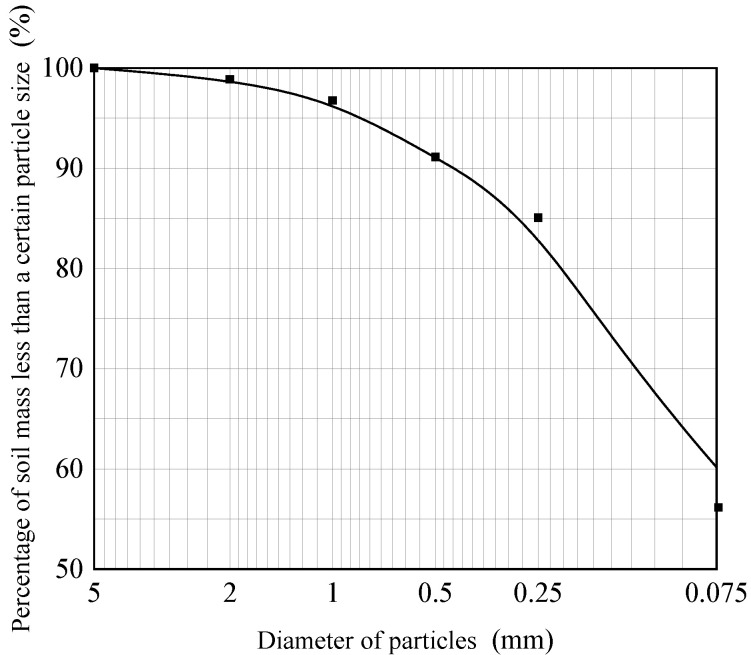
Particle-size distribution curve.

The soil samples were obtained from the vicinity of the Kaifeng City Wall. The physical properties are listed in Table 1, and the particle-size distribution curve is shown in Figure 2. Lime was purchased from Tianjin Kemiou Chemical Reagent Co., Ltd. (Tianjin, China). The main physical and chemical indexes were as follows: the relative molecular weight was 56.08, the CaO content was ≥98% and the pH value of sodium methylsilicate was between 12 and 14.

The collected silty clay was dried, crushed, and screened for later use. The compaction test was performed for the soil mixed with 7% lime. The maximum dry density of the silty clay is 1.68 g·cm^3^, and the optimal water content is 14.32%. The maximum dry density of the imitation ruins mixed with 7% lime is 1.608 g·cm^3^, and the optimal water content is 17.55%.

### 2.2. Sample Preparation 

The silty clay, 2% sodium methylsilicate, 7% lime, and pure water were mixed evenly and sealed. The mixture was allowed to stand for 24 h to promote the complete mixing of the silty clay, sodium methylsilicate, lime, and pure water. After standing, samples of size 39.1 mm × 80 mm were placed in an environment with different CO_2_ concentrations for carbonation curing and cured for 6 h, 12 h, 24 h, 48 h, 72 h, and 120 h. The different curing conditions are listed in Table 2. In addition, the pure soil group was set aside as the control group, and the silty clay and pure water were configured according to the water content of 17.55%. There were 25 test groups, and three parallel samples were set for each test group.

### 2.3. Experimental Method

1. Water-resistance test: in accordance with the standard for geotechnical test methods (GB/T50123-2019), a double-cup disintegration instrument was adopted (BJ-2 by Tianjin Tiangaung Optical Instruments Limited) and the sample quality was weighed during the test. The outer layer of the instrument was made of plexiglass, and the inner layer was composed of a beaker, wire mesh (10 cm diameter), ring knife (6.18 cm diameter), and steel wire. The sample was placed on a hanging net and then immediately placed into the beaker. The sample wetting at each stage was recorded in real-time using a recording device for 24 h.
2. Unconfined compressive strength (UCS) test: The specimens were dried in an oven at 105 °C for 48 h after curing and then subjected to the UCS test. The full-automatic geotechnical triaxial compression test system (TSZ-10) was used with a loading rate of 0.5 mm/min. When the pressure displacement curve in the test reached the peak value, the pressure reduced continuously for a period of time, after which the test was stopped, the peak pressure was recorded, test data were calculated, and a stress–strain relationship curve of the improved, repaired soil was obtained.
3. Soil Sample pH value test: A Shanghai Sanxin PH5S soil pH sensor was used to test the pH value under different curing conditions and different curing times. The changes in the pH of the soil were observed, and the influence of the carbonation reaction on the performance of the imitation earthen archaeological site was analyzed.
4. SEM test: SEM tests were performed using FEI Quanta 250 (Environmental Scanning Electron Microscope), USA. The crushed specimens were dried and cut into 0.5 cm × 0.5 cm × 0.5 cm soil blocks. The development of the pore space in the soil with was observed after the gold spray treatment to analyze the causes of macroscopic property changes from a microscopic perspective.

## 3. Experimental Results and Analysis

### 3.1. Effect of Curing Conditions on the Water-Resistant Performance of Imitation Earthen Archaeological Soil

**Figure 3:**
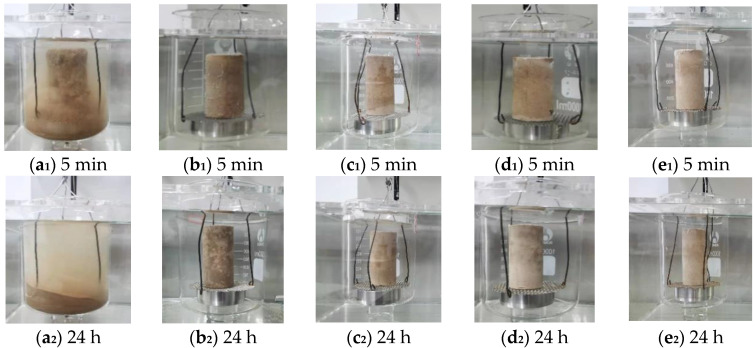
Water-resistance performance test ((**a**): pure soil; (**b**): 0.03%; (**c**): 5%; (**d**): 10%; (**e**): 15%).

To further observe the water-resistant performance of the imitation earthen archaeological site in depth, the pure soil and the imitation earthen archaeological site maintained in an environment with different CO_2_ concentrations were maintained for 120 h, The water-resistance test was performed after drying, and the influence of the carbonation reaction on the performance of the imitation earthen archaeological site was compared and analyzed. The pure soil group (Figure 3(a_1_,a_2_)) underwent complete wetting and disintegration within 5 min and could not be weighed for mass. For the specimens maintained in 0.03% CO_2_ concentration (consistent with the CO_2_ concentration in air) environment (Figure 3(b_1_,b_2_)), during the initial stage of the water-resistance test (Figure 3(b_1_)), the specimens exhibited no obvious wetting disintegration phenomenon, and tiny bubbles on the surface were observed along with a small amount of water absorption. After 24 h of the water-resistance test, (Figure 3(b_2_)), the color of the specimen changed because sodium methylsilicate did not fully react with water and CO_2_, resulting in uneven water resistance of the specimen. For the specimens of carbonization conservation in a 5% CO_2_ concentration environment (Figure 3(c_1_,c_2_)), during the initial stage of the water-resistance test, (Figure 3(c_1_)) no bubble generation and no obvious water absorption phenomenon were observed, and there was a better water-resistance effect. After 24 h of the water-resistance test, (Figure 3(c_2_)) the color of the outer surface was uniform, and there was no trace of water absorption. For the specimens (Figure 3(d_1_,d_2_,e_1_,e_2_)) of intensive carbonization conservation in a 10% CO_2_ and 15% CO_2_ concentration environment, the water-resistance test resulted in a change in the appearance of the specimens, similar to that observed in the 5% CO_2_ concentration environment, with relatively uniform surface color and no obvious traces of water absorption. This is because, under a high CO_2_ concentration and an intensive carbonation environment, sodium methylsilicate can fully react with water and CO_2_ to generate a polysiloxane film that can enhance the water resistance of the specimen. The specific chemical reaction is as shown in Equations (1) and (2). Moreover, it can be initially concluded that the increasing CO_2_ concentration is beneficial for improving the water-resistance effect of the imitation site soil.

2CH_3_Si(OH)_2_Ona + CO_2_ + H_2_O → 2[CH_3_Si(OH)_3_] + Na_2_CO_3_ (1)

n[CH_3_Si(OH)_3_] → [CH_3_Si(OH)_3/2_]n + 3/2 H_2_O (2)

**Figure 4:**
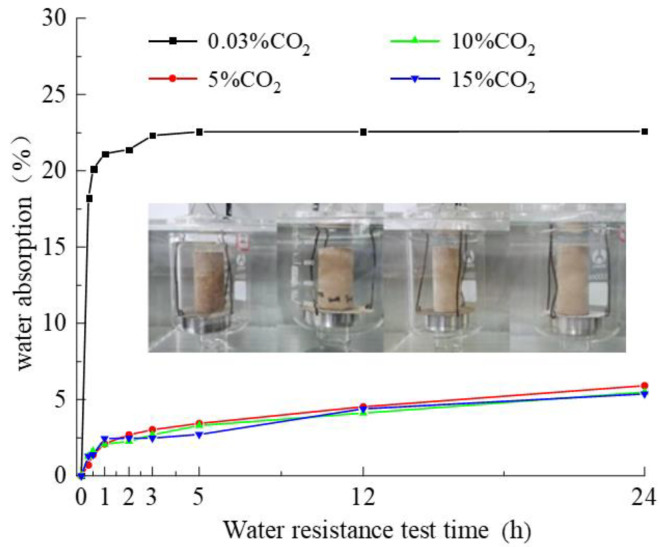
Effect of water absorption time on the water absorption rate of the specimen conditions.

The effect of water absorption with time on the water absorption rate of the specimens is shown in Figure 4. The water absorption rates of the specimens at 0.03%, 5%, 10%, and 15% CO_2_ concentrations were 22.59%, 5.92%, 5.49%, and 5.37%, respectively, after 24 h of specimen water absorption. The water absorption rates of the specimens maintained at 5%, 10%, and 15% CO_2_ concentrations were similar and decreased by 16.67%, 17.1%, and 17.22%, respectively, compared with those maintained at 0.03% CO_2_ concentration. This indicates that the water absorption of the imitation site soil does not decrease linearly with the increase in the CO_2_ concentration, and the water resistance of the imitation site soil can be improved by carbonization under 5% CO_2_ concentration. Maintaining proper carbonation can rapidly improve the water resistance of the imitation site soil and prevent the imitation site soil from collapsing when it is washed by rain.

**Figure 5:**
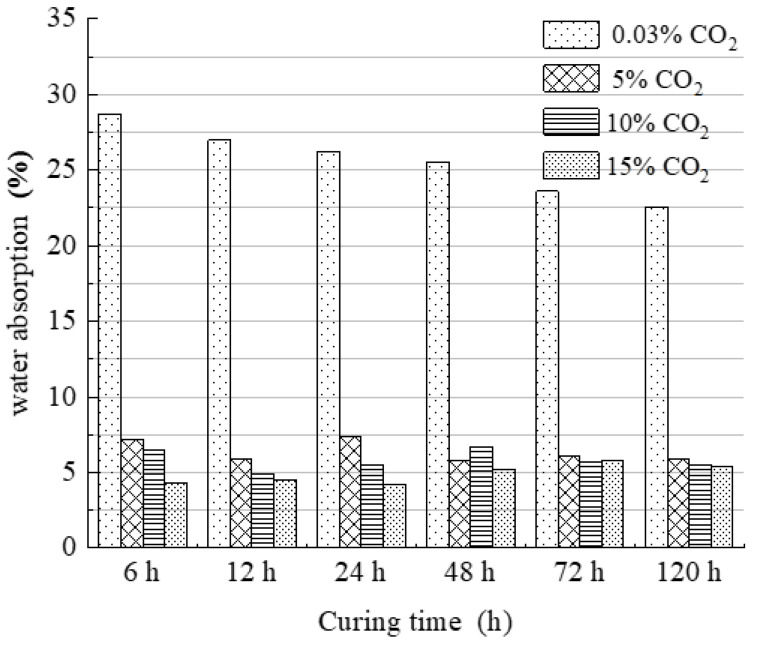
Curing time on the water absorption of the specimens.

The effect of time conservation on the water absorption of the specimens is shown in Figure 5. The water absorption of the specimens at 5%, 10%, and 15% CO_2_ concentrations under the same maintenance time was reduced to a greater extent than that of the specimens at 0.03% CO_2_ concentration. The water absorption of the specimens maintained at 5%, 10%, and 15% CO_2_ concentrations did not differ much under the same maintenance time. The imitation site soil could achieve better water resistance after 12 h of maintenance at 5% CO_2_ concentration because the addition of sodium methylsilicate forms a polysiloxane film on the surface of the specimen [23], and the imitation site soil has a strong water repellency. In addition, the CO_2_ concentration (5%, 10%, and 15%) had little effect on the water absorption of the specimens when the specimens were cured for more than 48 h. This may be because the carbonation reaction of sodium methylsilicate is completed after the specimens are maintained for 48 h at a 5% CO_2_ concentration. The increase in the CO_2_ concentration at this point has a limited influence on the water-resistance performance.

### 3.2. Effect of Carbonation on the Unconfined Compressive Strength of the Imitation Earthen Archaeological Site

**Figure 6:**
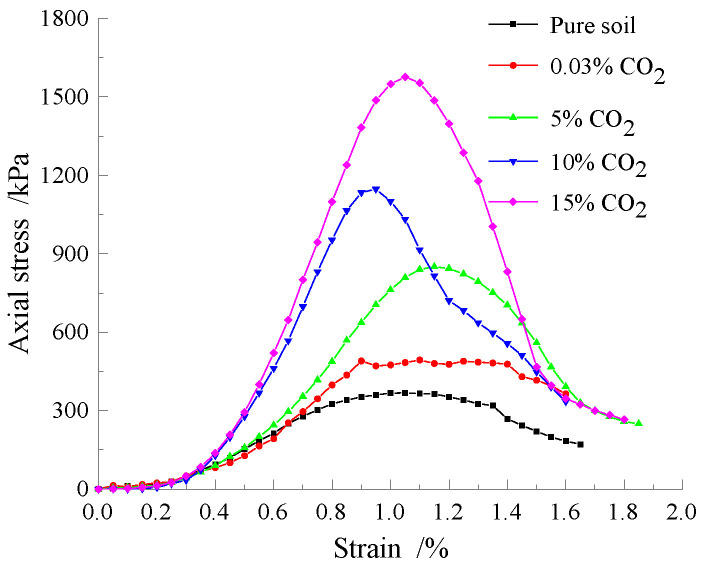
Stress–strain curves of the simulated site soil under different CO_2_ concentration curing conditions.

The effect of curing time on the UCS of the specimens is shown in Figure 6. The UCS of the imitation site soil gradually increased with the increase of the CO_2_ concentration in the conservation environment. After the specimens were maintained in the carbonation chamber at 0.03%, 5%, 10%, and 15% CO_2_ concentrations for 120 h, the UCS of the imitation site soil increased by 34.24%, 131.56%, 211.93%, and 329.29%, respectively, as compared to that of the plain soil. The UCS of the specimens at 5%, 10%, and 15% CO_2_ concentrations increased by 72.22%, 131.19%, and 219.27%, respectively, as compared to that of the specimens at 0.03% CO_2_ concentration.

**Figure 7:**
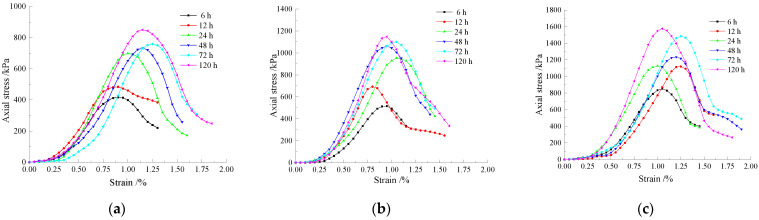
(**a**) Stress–strain curve of the simulated site soil with curing under 5% CO_2_ concentration. (**b**) Stress–strain curve of the simulated site soil with curing under 10% CO_2_ concentration. (**c**) Stress–strain curve of the simulated site soil with curing under 15% CO_2_ concentration.

The UCS increased with the increase in the curing time under the same CO_2_ concentration environment. Figure 7a–c indicates that the compressive strength increased by 16.19%, 34.27%, and 31.4% for the specimens maintained for 12 h as compared to those maintained for 6 h under 5%, 10%, and 15% CO_2_ concentration environments. The compressive strength of the 24 h-cured specimen increased by 44.21%, 37.68%, and 6.7%, respectively, compared with the 12-h specimen. The compressive strength of the cured 48-h specimen increased by 4.9%, 11.36%, and 9.49%, respectively, compared with the cured 24-h specimen. The compressive strength of the cured 72 h specimen increased by 3.59%, 3.58%, and 20.38%, respectively, compared with the cured 48-h specimen and 11.94%, 4.27%, and 6.09% for the specimens maintained for 120 h as compared to those maintained for 72 h. It can be seen that the UCS growth rate tends to increase and then decrease with the increase in the curing time. Moreover, the UCS growth rate is the highest at 24 h of curing time and then starts to decrease slowly.

Lime and sodium methylsilicate can effectively improve the compressive strength of powdered clay, and CO_2_ concentration and curing time have important effects on the UCS of the imitation site soil. A sufficient amount of CO_2_ accelerates the carbonation reaction inside the imitation site soil, and CaCO_3_ precipitation and Na_2_CO_3_ can be produced rapidly. The carbonate fills between soil particles, thereby enhancing the inter-particle bonding and increasing the strength of the imitation site soil. This law provides an important basis for optimizing the restoration process of the imitation site soil.

### 3.3. Effect of Carbonation on pH of Imitation Earthen Archaeological Site

**Figure 8:**
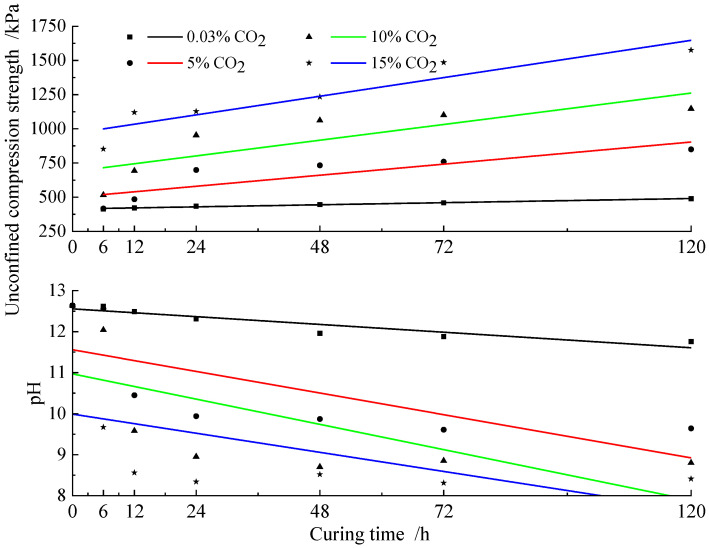
Relationship between pH value, unconfined compressive strength, and curing time.

Figure 8 shows the effect of the curing time on the pH of the specimens. With increasing curing time and CO_2_ concentration, the pH of the specimens gradually decreased and stabilized. Moreover, at the same CO_2_ concentration, the UCS and pH of the specimens exhibited opposite trends with the increase in the curing time, that is, the smaller the pH value, the larger the corresponding UCS. This was due to the conversion of strongly alkaline Ca(OH)_2_ to neutral CaCO_3_ deposition, during which the pH of the imitation site soil gradually decreased. Changes in pH values can reflect the state of CaCO_3_ deposition from the side and provide a basis for analyzing its mechanical properties [24]. The strength of the imitation site soil in the actual project can be preliminarily assessed from its pH value.

Under 5%, 10%, and 15% CO_2_ concentrations, the pH of the imitation site soil decreased significantly before 24 h and 12 h, and the growth rate of the compressive strength was also larger than that of other maintenance periods. However, after 24 h and 12 h, the pH of the imitation site soil and the growth rate of compressive strength decreased slowly. When Ca(OH)_2_ was partially converted to CaCO_3_ in the imitation site soil, the pH value of the imitation site soil was stabilized. This shows that increasing the CO_2_ concentration in the maintenance environment of the imitation site soil can effectively improve the carbonation reaction and the compressive strength of the imitation site soil. Moreover, it can accelerate the stabilization of the pH value of the soil, thereby improving the stability of the imitation site soil.

### 3.4. Microscopic SEM Analysis

To investigate the microscopic changes inside the mock site soils, the internal particle morphology and pore characteristics of the imitation site soil samples at different magnifications were analyzed using SEM. Soil specimens were analyzed in the pure soil group. Moreover, the soil specimens were maintained at 0.03% and 15% CO_2_ for 120 h to further investigate the mechanism of improving the water-resistant performance and unconfined compressive strength of the imitation site soil under different maintenance conditions.

**Figure 9:**
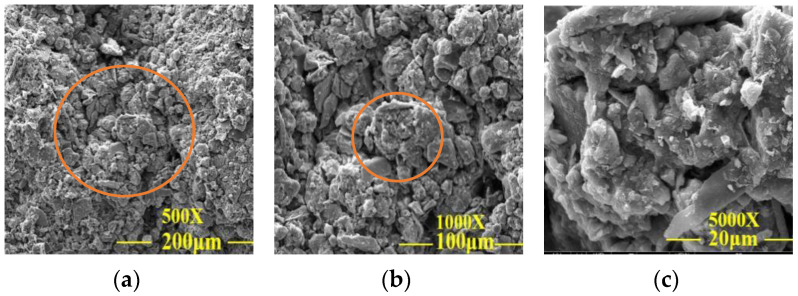
SEM images of the plain soil test group. (**a**) SEM analysis at a magnification of 500×; (**b**) SEM analysis at a magnification of 1000×; (**c**) SEM analysis at a magnification of 5000×.

**Figure 10:**
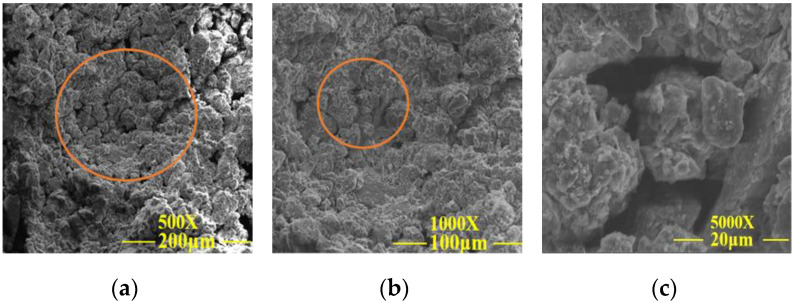
SEM images of the test group 6. (**a**) SEM analysis at a magnification of 500×; (**b**) SEM analysis at a magnification of 1000×; (**c**) SEM analysis at a magnification of 5000×.

**Figure 11:**
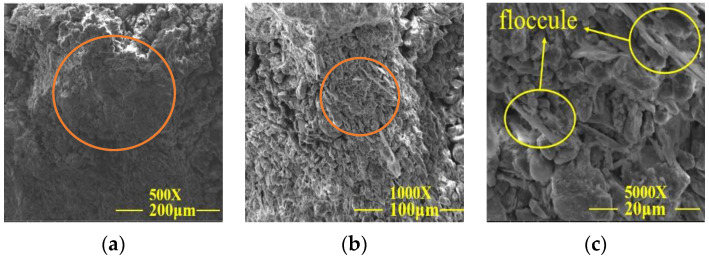
SEM images of the test group 24. (**a**) SEM analysis at a magnification of 500×; (**b**) SEM analysis at a magnification of 1000×; (**c**) SEM analysis at a magnification of 5000×.

Figure 9a–c shows that the internal porosity of plain soil was large, the arrangement of the soil particles was loose, and the particle grading was poor, resulting in weak adhesion between particles and that on the surface of soil particles. It can be seen from group 6 samples in Figure 10a–c that lime, sodium methylsilicate, and reaction products filled the gap between soil particles, improved the integrity of the soil skeleton, densified the filling between soil particles, enhanced the particle gradation, improved the adhesion between particles, reduced the porosity of the test sample, and effectively improved the disintegration resistance and unconfined compressive strength of the samples. From the 24 sets of samples in Figure 11a–c, it can be seen that the gap between the soil particles was small. Silicate gel particles in sodium silicate filled the pores of silty clay. The Ca (OH)_2_ and CaCO_3_ crystals generated from lime undergo a chemical reaction in the soil to make the dispersed soil particles cohesive into a whole. The deposition of CaCO_3_ can enhance cementation between soil particles, to improve the overall stability of the earthen archaeological site. The site imitation earthen archaeological site cured at 15% CO_2_ concentration can quickly generate CaCO_3_ crystals in 12 h curing time, further improving the disintegration resistance and unconfined compressive strength of the site imitation earthen archaeological site.

## 4. Conclusions

In this study, the Kaifeng City Wall was selected as the research object, and lime and sodium methylsilicate were selected as enhanced materials to prepare the Kaifeng City Wall imitation earthen archaeological site. The properties of the imitation earthen archaeological site were analyzed with test indices such as the specimen water absorption rate, compressive strength, microanalysis, and pH change of the imitation site soil. The following results were obtained:

1. The effect of carbonization maintenance on improving the water resistance of the imitation site soil was significant. Moreover, the imitation site soil can achieve better water resistance through carbonization maintenance under 5% CO_2_ concentration with the same maintenance time.
2. Carbonation curing has an obvious effect on improving the compressive strength of imitation earthen archaeological sites. The compressive strength of the samples cured in different CO_2_ concentration environments increased with the increase in CO_2_ concentration. Under the same CO_2_ concentration environment, the compressive strength increased with increasing curing time. The growth rate of the unconfined compressive strength was large with the curing time of 24 h, and then it decreased slowly.
3. Under the same curing conditions, the pH value of the imitation earthen archaeological site first decreased with increasing curing time and then gradually stabilized. Under the same carbonization curing time, the pH value of the imitation earthen archaeological site decreased with increasing CO_2_ concentration. The compressive strength showed a negative correlation with the pH value, that is, the smaller the pH value, the greater was the corresponding compressive strength. The interior of the soil sample was observed through SEM, which showed that the improved soil was densely filled among soil particles, the particle gradation was improved, and the adhesion between the particles was good. Moreover, we found that the porosity of the soil sample was reduced, the voids were less and dense, and CaCO_3_ precipitates were formed, which effectively improved the overall stability of the earthen archaeological site.

Increasing the CO_2_ concentration in the maintenance environment can make the carbonation reaction of the imitation earthen archaeological site uniform and rapid, promote CaCO_3_ precipitation, and effectively improve the specimen water absorption rate and compressive strength of the imitation site soil. Moreover, it can enhance the overall stability of the imitation earthen archaeological site.

## Figures and Tables

**Table 1:** Physical Properties of Silty Clay.

Natural Moisture Content/%	Plastic Limit/%	Liquid Limit/%	Plasticity Index	Maximum Dry Density/(g·cm^3^)	Optimum Water Content/%
13.2	21.03	37.63	16.6	1.68	14.32

**Table 2:** Different Conservation Environments of the City Wall Restoration Soil.

CO_2_ (%)	Curing Time (h)	Gro
0.03 (in air)	6	1
	12	2
	24	3
	48	4
	72	5
	120	6
5	6	7
	12	8
	24	9
	48	10
	72	11
	120	12
10	6	13
	12	14
	24	15
	48	16
	72	17
	120	18
15	6	19
	12	20
	24	21
	48	22
	72	23
	120	24
control group	120	25

## Data Availability

The data presented in this study are available on request from the corresponding author.
